# Diffuse infantile hepatic haemangioma and consumptive hypothyroidism: a clinical case with anaemia that raises suspicion

**DOI:** 10.1186/s13052-025-02027-2

**Published:** 2025-07-01

**Authors:** Giovanni Corsello, Elisa Costantini, Marco Sciveres, Adele Figuccia, Maria Cristina Maggio

**Affiliations:** 1 University Department PROMISE “G. D’Alessandro”, University of Palermo, Via del Vespro 129, Palermo, 90100 Italy; 2 Hepatology and Liver Transplant Unit IRCCS Ospedale Pediatrico Bambino Gesù, Roma, Italy

**Keywords:** Case report, Infantile hepatic haemangiomas, Consumptive hypothyroidism

## Abstract

**Background:**

Infantile hepatic haemangiomas are benign liver tumours, with growth and regression phases of the tumour, corresponding to those of infantile cutaneous haemangiomas. The classification and the pathogenesis need further insights. Though most infantile hepatic haemangiomas are asymptomatic, some patients show severe complications, such as high-output congestive cardiac failure, anemia, thrombocytopenia, consumptive coagulopathy, liver failure and consumptive hypothyroidism. A fatal clinical evolution is described in some patients. The heterogeneity of the lesion’s diffusion and of the disease-related comorbidities make the treatment challenging. The treatment with oral propranolol is effective and allows symptoms regression.

**Case presentation:**

We report the case of a two-month-old female with the first diagnosis of late-onset congenital hypothyroidism, associated to unexplained anemia and significant increase of transaminases and gamma-GT. She promptly started treatment with levothyroxine (10 mcg/kg/day). To identify the etiology of hypothyroidism, anemia and increased liver enzymes, she underwent an abdominal ultrasound, that evidenced infantile diffuse hepatic hemangiomatosis, confirmed by abdominal MRI. Brain MRI showed a few millimetric areoles, compatible with microangiomas. The patient needed a significant increase of levothyroxine dosage, reaching a difficult normalization of TSH, fT4 and fT3. Ten days after the start of treatment with propranolol, a significant reduction in liver hemangiomatosis occurred, confirmed by the reduction of alpha-fetoprotein, AST, ALT, gamma -GT and TSH levels. The patient required a progressive reduction of levothyroxine dose, with the improvement of hematologic parameters. The child’s auxological growth and neuromotor development occurred in an age-appropriate manner.

**Conclusions:**

In the case described hereby, complications such as anemia, hypothyroidism, hepatomegaly, and impaired liver function, required to start therapy with propranolol, with the improvement of clinical and laboratory parameters. High-dose levothyroxine replacement therapy is mandatory to preserve the neurological development that occurs when hypothyroidism is inadequately treated throughout the proliferative phase of haemangiomas. In fact, the prognosis is strongly determined by the early identification of haemangiomatosis as the cause of hypothyroidism and of the other complications. Systemic impairment in early phases may be very subtle, requiring a prompt diagnosis and a multidisciplinary approach to undertake appropriate therapy and prevent short- and long-term sequelae.

## Background

Infantile hemangiomas (IHs) are the most common pediatric vascular tumors that predominantly affect females and have an incidence of 4–5% in full-term infants [1, 2]. IHs are benign tumors that are characterized by a rapid proliferative phase during the first 6–10 months, followed by a slow involution phase, that may last up to 10 years [3]. They may involve skin and organs such as the liver and the brain. Risk factors include low birth weight, prematurity and positive family history.

Infantile Hepatic haemangiomas (IHHs), are indeed the most common liver tumors in childhood [4] and, depending on the number of lesions, they are classified into focal IHHs, multifocal IHHs, and diffuse IHHs [5]. Despite they are usually asymptomatic, IHHs may be accompanied by hypothyroidism, anemia, thrombocytopenia, coagulopathy, liver failure and heart failure, which require prompt medical treatment. In fact, according to the classification by Meyers RL. et al. IHHs are classified by number of lesions into the following subtypes:

- **Focal**: single liver lesion, typically asymptomatic and rarely associated with cutaneous IH, sometimes associated with anemia and thrombocytopenia.
- **Multifocal**: multiple distinct lesions in the liver parenchyma that may be associated with moderate cardiomegaly and high output heart failure from arteriovenous and porto-venous shunt; in 60% of cases, multifocal lesions are accompanied by cutaneous counterparts.
- **Diffuse**: replacement of a large part of the liver parenchyma with various proliferating lesions of angiomatous nature; cutaneous IH may be present; may be associated with high output cardiac failure from arteriovenous and porto-venous shunts, severe hepatomegaly with compartment syndrome, respiratory distress and severe hypothyroidism [5].

Abdominal ultrasound is recommended to assess the presence of hepatic hemangiomas in any child under the age of six months presenting five or more cutaneous hemangiomas [6].

The definitive diagnosis of IHs could be made by biopsy and subsequent histopathological examination.

However, considering that these highly vascularized lesions have a high risk of bleeding, biopsy is rarely performed. Hence, radiographic findings and laboratory investigations are needed to the diagnosis and to exclude malignant processes.

Lesions must be monitored by serial ultrasound scans because a lesion that grows rapidly from birth until about eight months of age and then begins to regress is compatible with IHs [2].

Although IHs are usually considered benign lesions, a close follow-up should be planned in the case of liver lesions. In fact, systemic complications can be associated with these lesions [7]. Furthermore, specific attention should be paid to symptoms like growth retardation, thyroid dysfunction, cardiac dysfunction and feeding difficulties, which may be signs of systemic impairment [8].

Furthermore, an uncommon yet concerning condition that may be associated with diffuse IHHs is secondary consumptive hypothyroidism secondary to higher expression of the iodothyronine deiodinase type 3 enzyme (D3) in the hemangioma tissue. The enzyme is normally expressed in the placenta and in the brain and leads to an increased degradation of thyroxine to reverse triiodothyronine and the conversion of triiodothyronine to 3,3’-diiodothyronine, both biologically inactive forms [9].

These patients develop hypothyroidism in neonatal age, mimicking a picture of congenital hypothyroidism (CH) [10]. Thyroid hormones are key of central nervous system development, maturation, and myelination. Additionally, hypothyroidism affects growth and the function of several systems, including the musculoskeletal system, as demonstrated by studies on psychomotor abilities and posture [11]. Early treated patients with CH show normal muscle strength. However, these patients show poor postural control ability, indicating the role of thyroid hormones in the adequate development of children, since the first stage of their life [11].

Hence, considering that the proliferative phase of hemangiomas corresponds to the critical period of neurodevelopment, the condition may lead to serious complications that require prompt interventions.

For these reasons, screening is essential to promptly start therapy of hepatic hemangiomas, using propranolol in combination with aggressive treatment for hypothyroidism, which can require very high doses of L-T4.

We hereby report the case of a two-month-old infant diagnosed with IHs with multiple skin and liver localizations associated with hypothyroidism, anemia and massive alterations of the liver parenchyma.

The aim of our work is to describe a clinical case characterized by rarity, diagnostic and therapeutic complexity, to make available and share our experience. Sharing rare and complex cases is an indispensable contribution to the care of these patients.

## Case presentation

**Fig. 1:**
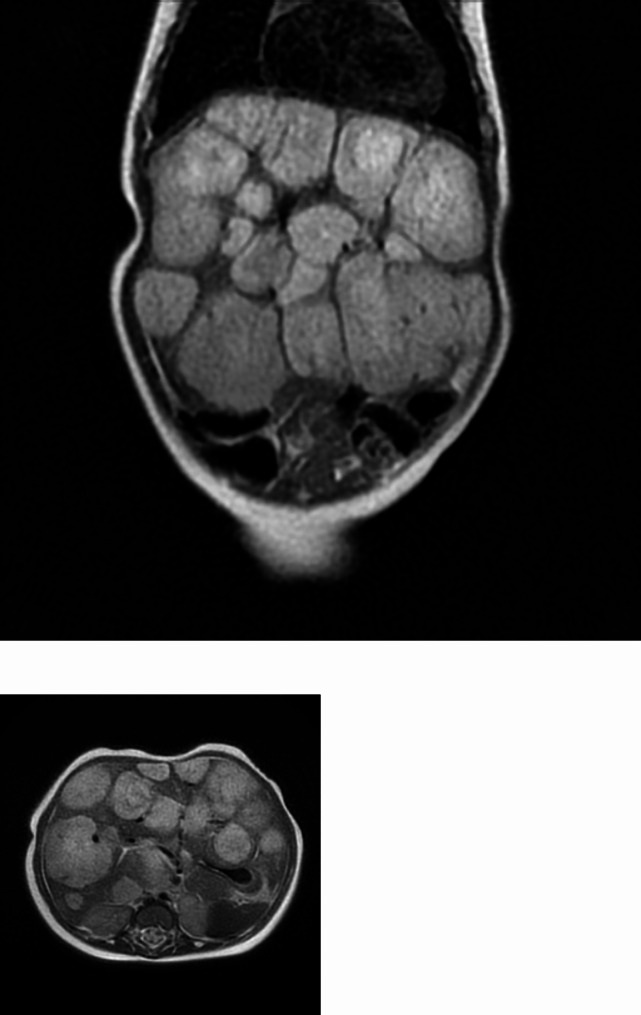
MRI of abdomen at the diagnosis of diffuse infantile hepatic haemangioma

We describe the clinical case of a 2-month-old female infant. She is firstborn, delivered at 40 + 3 weeks of gestation. At birth: weight 3060 g, Apgar 1 min: 9, 5 min: 10, good adaptation to extrauterine life. She received exclusive breastfeeding. Anamnestic records reported that the mother had autoimmune hypothyroidism treated, during pregnancy, levothyroxine. Metabolic screening and neonatal screening for CH were normal. At two weeks of life, TSH, fT4 and fT3 were detected for the anamnestic record of autoimmune thyroiditis of the mother. In this occasion, high TSH values (7.98 mUI/L), with normal values of fT3 e fT4 were evidenced, in the absence of clinical signs of hypothyroidism. One month later, rising TSH values (18.28) with antibodies (Ab anti-TPO, Ab anti-TG, and Ab anti-TSH receptor) in the normal range were detected (see Table 1). Therefore, replacement therapy with levothyroxine was started for suspected CH. However, the relief of anemia, with hemoglobin value of 8 g/dl, required further hematologic investigations. Five days later, due to the appearance of pale skin, a blood sample was taken, with the findings of: Hb: 7.2 g/dl, AST: 95 U/L, ALT: 31 U/L (Table 1). The patient was hospitalized for the necessary diagnostic tests. The objective examination evidenced: pale skin, three flat cutaneous angiomas in the glabellar area, on the left auricle and in the nape; a systolic murmur 1/6 L, globular abdomen, significant hepatomegaly, no sign of cardiac decompensation. Coombs test and the study of pathological hemoglobins were negative. Blood transfusion was performed. Examinations for metabolic and infectious causes were made; the abdominal ultrasonography evidenced an enlarged and diffusely inhomogeneous pseudo nodular liver, and the abdominal Magnetic Resonance Imaging (MRI) revealed several expansive lesions of variable diameter size, from 7 mm up to 70 mm, compression of the portal vein and splenic vein, dilatation of the hepatic proper artery and common hepatic artery, dislocation of the coeliac tripod (Fig. 1). The MRI was compatible with “multifocal/diffuse haemangiomatosis”. Transfontanellar ultrasound, eye examination and brain MRI were performed to rule out PHACE(s) syndrome [12]. However, brain MRI permitted the finding of a few millimetric areoles of clear hypointensity in the diffuse T2* sequence, compatible with microangiomas. Cardiac ultrasonography showed patent oval foramen, without any morpho functional changes and no signs of overload. Blood examinations showed a rapid increase of: AST 183 U/L, ALT 75 U/L, gamma-GT: 540 U/L (n.v. 8–90 U/L). Other blood examinations evidenced: total bilirubin 1.65 mg/dl, direct 0.66 mg/dl, indirect 0.99 mg/dl, LDH 469 U/L, alpha-fetoprotein 25384 ng/ml (40-1000 ng/ml), total protein 6.1 g/dl, albumin 4.4 g/dl, normal coagulation parameters, further increase of TSH levels, requiring the increase of L-thyroxine dose. During hospitalization, TSH values gradually increased (maximum value 129 mUI/L), requiring the levothyroxine dose increase (see Table 1). In parallel, an increase in hepatomegaly was noted, both on clinical examination and with ultrasound evaluation. In addition, AST, ALT, gamma-GT progressively increased, with parallel reduction of Hb.

**Table 1:** Hormonal and hepatic enzymes values of the infant during the follow-up

Age	14 days	45 days	50 days	60 days	74 days	78 days	105 days
TSH (mUI/L)	7.98	18.28	92.96	129	39.2	18.1	0.13
fT4 (pg/ml)	2.22	1.8	1.53	1.6	1.7	1.97	2.57
fT3 (ng/dl)	6.23	3.26	3.37	2.99	2.45	1.9	2.33
AST (U/L)		95	183	134	183	157	89
ALT (U/L)		31	75	41	75	111	91
gamma-GT (U/L)		542	566	733	448	391	149
Bilirubin (total/direct) (mg/dl)		1.13/0.31	1.65/0.66	1.01/0.6	1.07/0.46	0.65/0.36	0.42/0.11
Hb (g/dl)		8	7.2	10.5		11.3	10.5
Platelet count (x10^3^/mcl)		487,200	636,100	616,000	247,300	443,000	616,000
Alpha-fetoprotein (40-1000 ng/ml)			25,384	25,334		11,015	2444
Treatment with L-thyroxine (mcg/day)		37.5	50	75	75	100 for 3 days/week; 75 for 4 days/week	75

Laboratory investigations such as the outcome of blood values of alpha-fetoprotein (see Table 1) and negative urinary catecholamines were useful in ruling out hepatoblastoma or neuroblastoma.

MRI imaging, the presence of angiomas in other locations allowed the differential diagnosis with Kaposiform hemangioendothelioma (KHE) with Kasabach-Merrit phenomenon, undifferentiated embryonal sarcoma and angiosarcoma [2], and benign lesions such as cysts, biliary hamartomas and arteriovenous malformations [5].

Based on the current guidelines [13], we started propranolol therapy under continuous monitoring of vital parameters (heart rate, blood pressure and blood glucose levels) and boosted up to a dosage of 3 mg/kg/day in the absence of clinical-ECG and echocardiographic changes.

Ten days after starting propranolol therapy, TSH concentration began to decrease and at 3.5 months of life, TSH levels were suppressed (TSH 0.125 mUI/L, fT3: 2.33 ng/dl, fT4: 2.57 pg/ml). Hence, the levothyroxine dose was gradually reduced (Table 1).

Simultaneously, after ten days of treatment with propranolol, abdominal ultrasound showed a significant reduction (12%) of a target lesion (4.3 cm VS 3.8 cm). Four months later, the longest diameter of the same lesion decreased to 1.7 cm. A partial reduction of liver cytolysis enzymes and of alpha feto-protein values was observed.

The control of the hypothyroidism was simultaneous with the reduction of the diffuse childhood hepatic angiomatosis; this supported the diagnosis of consumptive hypothyroidism.

The patient’s follow-up showed a gradual and progressive reduction of levothyroxine dose, with a good control of hypothyroidism. When the child was 10 months old, the volume of the angiomas was significantly reduced, but not wholly resolved. The weight was 8,550 gr (55th Centile) and the length was 69 cm (28th Centile). The dose of levothyroxine prescribed was 12.5 mcg/day. The neuromotor development occurred in an age-appropriate manner.

## Discussion and conclusions

In the case described above, the rapid extension and progression of hepatic haemangiomatosis and the initial non-response to replacement therapy with levothyroxine were the main clinical challenges.

In this patient the diagnosis is “diffuse infantile hemangiomatosis”, a subtype of IH that is associated with the highest risk of morbidity and mortality, secondary to massive replacement of liver parenchyma with voluminous hemangiomas. This subtype is related to the rare condition of consumptive hypothyroidism first described in 2000 by Huang et al. [9].

Severe hypothyroidism occurs due to overexpression of D3 in the haemangioma, a selenium-enzyme physiologically localised in the central nervous system, in placenta and in fetal liver, which catalyzes the conversion of T4 to reverse T3 and T3 to 3,3’-diiodothyronine, both biologically inactive forms; replacement therapy may require very high doses of levothyroxine until the angiomas regress.

Consumptive hypothyroidism is characterized by low free T3 (fT3), normal or low free T4 (fT4) and high TSH levels. Patients have elevated serum rT3 levels due to increased degradation of T4 and T3 by D3. The high D3 levels in IH are not fully understood but may be related to the histologic similarity between the endothelial cells of hemangiomas and the placenta, which share certain immunohistochemical markers such as GLUT-1. Probably IH originated from placental angioblasts with which they share self-limiting growth and high D3 activity [8]. In childhood hypothyroidism, like in our case, which is not responsive to replacement therapy, it is necessary to consider alternative forms of hypothyroidism, and exclude atypical causes of primary hypothyroidism (iodothyronine resistance, non-thyroidal illnesses and consumptive hypothyroidism).

Consumptive hypothyroidism is a rare condition and approximately 52 cases have already been described in literature [14]. Suspicious elements are the rapid increase in TSH with low fT3, despite replacement therapy, the association with anaemia, increased AST, ALT, gamma-GT. We recommend abdominal ultrasound in the presence of 5 or more cutaneous haemangiomas. However, IHHs can also be found in patients with one or no cutaneous haemangioma. Periodical thyroid function assessment is necessary in patients with hepatic haemangiomas, particularly when lesions’ size and number increase rapidly.

However, in our patient, as in other cases described in the literature, neither the number of cutaneous angiomas nor their morphological appearance were elements of diagnostic suspicion of diffuse IHHs. Hence, we stress that the diagnostic procedure by ultrasound screening is to be considered in cases with a smaller number of cutaneous haemangiomas, if initial signs of systemic impairment are reported or in newborns with CH and unexplained anemia.

In our case, the diagnosis of IHH was made by complete physical examination (three flat angiomas, marked skin pallor and significant hepatomegaly), blood tests (severe anemia and elevated alpha-fetoprotein values) and diagnostic imaging; abdomen ultrasound showed the presence of an enlarged and diffusely inhomogeneous pseudo nodular liver that has been confirmed by abdominal MRI, and led to a clinical picture that was compatible with multifocal/diffuse childhood haemangiomatosis.

In most cases, angiomas tend to be resolved without intervention. Close follow-up is necessary; 80% of all haemangiomas reach their final size within three months of life [1].

In the case described the onset of complications such as anemia, hypothyroidism, hepatomegaly with impaired liver function, was present since the time of diagnosis. It was necessary to progressively and rapidly increase the dose of levothyroxine, and Propranolol was started with an increasing dose up to 3 mg/Kg/day. Propranolol is a beta-blocking, non-selective drug that inhibits B1 (primarily myocardial) and B2 receptors (present in adipose tissue, pancreas, liver, smooth muscle). The exact mechanism underlying efficacy in IHH is not yet fully understood. However, the immediate effects could be attributed to vasoconstriction, the intermediate effects might be related to reduced expression of VEGF and FGF, and finally the long-term effects could be interpreted as being the result of apoptosis of capillary endothelial cells.

Probably propranolol causes suppression of angiogenic factors through suppression of the production of cyclic adenosine monophosphate and other angiogenic components such as metalloproteinases, endothelial growth factors and fibroblast growth factors [1, 15–16].

According to guidelines, propanol can be administered in children aged between 5 weeks and 5 months for a duration of at least 6 months [13, 17–19].

The initial dose is 0.5-1 mg/kg/day, divided into two daily doses, and can be gradually increased to a maximum dosage of 3 mg/kg/day. Possible side effects are bradycardia, hypotension, hypoglycemia, lethargy, and difficulty in sucking [20].

In our case, in the absence of initial side effects, therapy was continued at home, titrating the dose according to weight gain.

Combination therapy with propranolol and levothyroxine was monitored by blood tests that included transaminases, bilirubin, coagulation factors, TSH, fT4 and fT3.

According to the literature, we observed that a good control of hypothyroidism was achieved with the involution of IHH [2, 19].

Thyroid hormones, alpha-fetoprotein levels and liver cytolysis indexes appeared to be excellent biomarkers of propranolol response. The improvement in the patient’s clinical and laboratory conditions and the reduction of the target lesion on ultrasound, showed how effective propranolol treatment can be even in this type of haemangioma.

In combination with propranolol, high-dose levothyroxine replacement therapy is necessary to preserve the neurodevelopment steps of the child, that can be compromised during the proliferative phase of haemangiomas. A specialistic endocrinologist follow-up is necessary, to prevent irreversible growth and intellectual retardation. Furthermore, considering the refractory nature of this specific form of hypothyroidism, it is sometimes necessary to switch rapidly to doses of levothyroxine that are higher than usual so to minimize the risk of long-term sequelae [8]. 

The case described underlines the importance of a timely diagnosis, early therapy and a multidisciplinary approach in the management of these patients. The short- and long-term prognosis, also in terms of neuromotor development, is strongly determined by the early identification of haemangiomatosis as the cause of hypothyroidism, cardiac dysfunction and hematologic complications.

## Data Availability

The datasets used and/or analysed during the current study are available from the corresponding author on reasonable request.

## Abbreviations

IHs: Infantile hemangiomas
IHHs: Infantile Hepatic haemangiomas
D3: Iodothyronine deiodinase type 3 enzyme
CH: Congenital hypothyroidism
MRI: Magnetic Resonance Imaging

## References

[1] Léauté-Labrèze C, Harper JI, Hoeger PH, Infantile haemangioma. Lancet. 2017;390(10089):85–94. 10.1016/S0140-6736(16)00645-0. Epub 2017 Jan 13. PMID: 28089471. 28089471 10.1016/S0140-6736(16)00645-0

[2] Lewis D, Vaidya R. Congenital and Infantile Hepatic Hemangioma. [Updated 2023 Jun 12]. In: StatPearls. Treasure Island (FL): StatPearls Publishing; 2025 Jan-. Available from: https://www.ncbi.nlm.nih.gov/books/NBK518988/30085530

[3] Itinteang T, Withers AH, Davis PF, Tan ST. Biology of infantile hemangioma. Front Surg. 2014;1:38. 10.3389/fsurg.2014.00038. PMID: 25593962; PMCID: PMC4286974. 25593962 10.3389/fsurg.2014.00038 PMC4286974

[4] Yang B, Li L, Zhang LX, Sun YJ, Ma L. Clinical characteristics and treatment options of infantile vascular anomalies. Med (Baltim). 2015;94(40):e1717. 10.1097/MD.0000000000001717. PMID: 26448027; PMCID: PMC4616746. 10.1097/MD.0000000000001717 PMC4616746 26448027

[5] Gnarra M, Behr G, Kitajewski A, Wu JK, Anupindi SA, Shawber CJ, Zavras N, Schizas D, Salakos C, Economopoulos KP. History of the infantile hepatic hemangioma: from imaging to generating a differential diagnosis. World J Clin Pediatr. 2016;5(3):273–80. 10.5409/wjcp.v5.i3.273. PMID: 27610342; PMCID: PMC4978619. 27610342 10.5409/wjcp.v5.i3.273 PMC4978619

[6] Dickie B, Dasgupta R, Nair R, Alonso MH, Ryckman FC, Tiao GM, Adams DM, Azizkhan RG. Spectrum of hepatic hemangiomas: management and outcome. J Pediatr Surg. 2009;44(1):125– 33. 10.1016/j.jpedsurg.2008.10.021. PMID: 19159729. 10.1016/j.jpedsurg.2008.10.021 19159729

[7] Rialon KL, Murillo R, Fevurly RD, Kulungowski AM, Christison-Lagay ER, Zurakowski D, Kozakewich HP, Alomari AI, Fishman SJ. Risk factors for mortality in patients with multifocal and diffuse hepatic hemangiomas. J Pediatr Surg. 2015;50(5):837–41. Epub 2014 Dec 5. PMID: 25783331. 25783331 10.1016/j.jpedsurg.2014.09.056

[8] Campbell V, Beckett R, Abid N, Hoey S. Resolution of consumptive hypothyroidism secondary to infantile hepatic hemangiomatosis with a combination of propranolol and Levothyroxine. J Clin Res Pediatr Endocrinol. 2018;10(3):294–8. 10.4274/jcrpe.4865. Epub 2018 Feb 28. PMID: 29537380; PMCID: PMC6083462. 29537380 10.4274/jcrpe.4865 PMC6083462

[9] Huang SA, Tu HM, Harney JW, Venihaki M, Butte AJ, Kozakewich HP, Fishman SJ, Larsen PR. Severe hypothyroidism caused by type 3 iodothyronine deiodinase in infantile hemangiomas. N Engl J Med. 2000;343(3):185-9. 10.1056/NEJM200007203430305. PMID: 10900278. 10.1056/NEJM200007203430305 10900278

[10] Maggio MC, Ragusa SS, Aronica TS, Granata OM, Gucciardino E, Corsello G. Neonatal screening for congenital hypothyroidism in an Italian centre: a 5-years real-life retrospective study. Ital J Pediatr. 2021;47(1):108. 10.1186/s13052-021-01053-0. PMID: 33952334; PMCID: PMC8097769. 33952334 10.1186/s13052-021-01053-0 PMC8097769

[11] Brusa J, Maggio MC, Giustino V, Thomas E, Zangla D, Iovane A, Palma A, Corsello G, Messina G, Bellafiore M. Upper and lower limb strength and body posture in children with congenital hypothyroidism: an observational Case-Control study. Int J Environ Res Public Health. 2020;17(13):4830. 10.3390/ijerph17134830. PMID: 32635579; PMCID: PMC7370191. 32635579 10.3390/ijerph17134830 PMC7370191

[12] Stănciulescu MC, Dorobantu FR, Boia ES, Popoiu MC, Cerbu S, Heredea R, Iacob ER, Cimpean AM, Caplar BD, Popoiu AV. Face(s) of a PHACE(S) syndrome patient before and after therapy: particular case report and review of literature. Child (Basel). 2022;9(12):1970. 10.3390/children9121970. PMID: 36553413; PMCID: PMC9776585. 10.3390/children9121970 PMC9776585 36553413

[13] Krowchuk DP, Frieden IJ, Mancini AJ, Darrow DH, Blei F, Greene AK, Annam A, Baker CN, Frommelt PC, Hodak A, Pate BM, Pelletier JL, Sandrock D, Weinberg ST, Whelan MA, Subcommittee on the management of infantile hemangiomas. Clinical Practice Guideline for the Management of Infantile Hemangiomas. Pediatrics. 2019;143(1):e20183475. 10.1542/peds.2018-3475. PMID: 30584062. 10.1542/peds.2018-3475 30584062

[14] Siano MA, Ametrano O, Barbato F, Sammarco E, Ranucci G, Pietrobattista A, Rossomando A, Mandato C. Consumptive hypothyroidism due to hepatic hemangiomas: A case series and review of the literature. JPGN Rep. 2022;3(4):e270. 10.1097/PG9.0000000000000270. PMID: 37168485; PMCID: PMC10158424. 37168485 10.1097/PG9.0000000000000270 PMC10158424

[15] Kum JJ, Khan ZA. Mechanisms of propranolol action in infantile hemangioma. Dermatoendocrinol. 2015;6(1):e979699. 10.4161/19381980.2014.979699. PMID: 26413184; PMCID: PMC4580045. 26413184 10.4161/19381980.2014.979699 PMC4580045

[16] Storch CH, Hoeger PH. Propranolol for childhood haemangiomas: insights into the molecular mechanisms of action. Br J Dermatol. 2010;163(2):269–74. 10.1111/j.1365-2133.2010.09848.x. Epub 2010 May 8. PMID: 20456345. 20456345 10.1111/j.1365-2133.2010.09848.x

[17] Léauté-Labrèze C, Hoeger P, Mazereeuw-Hautier J, Guibaud L, Baselga E, Posiunas G, Phillips RJ, Caceres H, Lopez Gutierrez JC, Ballona R, Friedlander SF, Powell J, Perek D, Metz B, Barbarot S, Maruani A, Szalai ZZ, Krol A, Boccara O, Foelster-Holst R, Febrer Bosch MI, Su J, Buckova H, Torrelo A, Cambazard F, Grantzow R, Wargon O, Wyrzykowski D, Roessler J, Bernabeu-Wittel J, Valencia AM, Przewratil P, Glick S, Pope E, Birchall N, Benjamin L, Mancini AJ, Vabres P, Souteyrand P, Frieden IJ, Berul CI, Mehta CR, Prey S, Boralevi F, Morgan CC, Heritier S, Delarue A, Voisard JJ. A randomized, controlled trial of oral propranolol in infantile hemangioma. N Engl J Med. 2015;372(8):735– 46. 10.1056/NEJMoa1404710. PMID: 25693013. 10.1056/NEJMoa1404710 25693013

[18] Hoeger PH, Harper JI, Baselga E, Bonnet D, Boon LM, Ciofi Degli Atti M, El Hachem M, Oranje AP, Rubin AT, Weibel L, Léauté-Labrèze C. Treatment of infantile haemangiomas: recommendations of a European expert group. Eur J Pediatr. 2015;174(7):855–65. Epub 2015 May 29. PMID: 26021855. 26021855 10.1007/s00431-015-2570-0

[19] Léaute-Labrèze C, Boccara O, Degrugillier-Chopinet C, Mazereeuw-Hautier J, Prey S, Lebbé G, Gautier S, Ortis V, Lafon M, Montagne A, Delarue A, Voisard JJ. Safety of Oral Propranolol for the Treatment of Infantile Hemangioma: A Systematic Review. Pediatrics. 2016;138(4):e20160353. 10.1542/peds.2016-0353. PMID: 27688361. 10.1542/peds.2016-0353 27688361

[20] Konrad D, Ellis G, Perlman K. Spontaneous regression of severe acquired infantile hypothyroidism associated with multiple liver hemangiomas. Pediatrics. 2003;112(6 Pt 1):1424-6. 10.1542/peds.112.6.1424. PMID: 14654623. 10.1542/peds.112.6.1424 14654623

